# Mother to child transmission (MTCT) of HIV - almost a thing of the past? A cohort study of HIV positive women starting antiretroviral drugs in pregnancy

**DOI:** 10.1186/1471-2334-14-S4-O6

**Published:** 2014-05-29

**Authors:** Hugh A Leonard, Ana Gonzalez, Lisa Burch, Neal Marshall, Fathulla Badenan, Debbie Levitt, Ana Garcia, Alison Wright, Rimi Shah, Daniel Webster, Margaret Johnson

**Affiliations:** 1 Department of Virology, Royal Free London NHS Foundation Trust, United Kingdom; 2 Department of Obstetrics and Gynaecology, Royal Free London NHS Foundation Trust, United Kingdom; 3 Research Department of Infection and Population Health, UCL, London, United Kingdom; 4 Ian Charleson Centre for HIV Medicine, Royal Free London NHS Foundation Trust, United Kingdom; 5 Clare Simpson Clinic for HIV Medicine, Barnet and Chase Farm Hospitals NHS Trust, United Kingdom

## 

Where available, antiretrovirals (ARVs) and antenatal HIV testing and care have significantly reduced mother to child transmission (MTCT). Higher maternal viral load (VL) is linked with higher risk of MTCT, increasing risk of MTCT for women starting ARVs during pregnancy compared to those already suppressed.

A single-centre, retrospective cohort of women starting ARVs during pregnancy from 2004 till 2013. Demographic, obstetric and virological data, and neonatal outcomes were collected where available.

60 pregnancies were recorded (in 56 women) in which ARVs were started or restarted from a total of 129 recorded pregnancies. 48% (27/56) were new antenatal HIV diagnoses and in these median gestational age (GA) at diagnosis was 16.1 weeks (range 5.3-37.6). Median GA at ARV commencement was 22.4 weeks (range 8-37.7) with 63% (35/56) starting before 24 weeks and 91% (51/56) before 28 weeks. HIV diagnosis during pregnancy was associated with a later commencement of ARVs (23.9 vs 19.9 weeks, p=0.009). The ARV regimen was available for 58 pregnancies. Treatment was with two NRTIs plus NNRTI (3), or PI (54) or raltegravir (1). Raltegravir was added as a forth agent in 7 patients.

87% (52/60) had resistance genotyping before (14) or during (38) pregnancy, 81% (22/27) for new diagnoses in pregnancy. The rate of any ARV resistance was 12% (6/52): 4 patients had NNRTI and 2 NRTI resistance mutations, 4 were treatment-naïve. This was not associated with treatment failure.

HIV VL at delivery was available in 57 pregnancies with detectable VL in 16% (9/57). Pre-treatment VL >100,000 copies/mL during pregnancy was associated with higher risk of detectable VL at delivery (p=0.016). 69% babies were delivered by Caesarean section, 32% as an emergency. There was one late miscarriage at 17 weeks. Median GA at birth was 38 weeks (range 17-42) with 21% born at <37 weeks (10/48). There was one HIV MTCT (1.7% for those starting ARV in pregnancy, 0.8% overall) in a woman who was poorly adherent with ARVs throughout pregnancy with a VL of 216 copies/mL at delivery following a period of directly observed therapy.

Over 10 years of integrated HIV and antenatal care, there was only one case of MTCT. Initiating ART for prevention of MTCT is complex, requires a multi-disciplinary approach and importantly patient engagement. Antenatal practices and guidelines have changed over the period of this dataset, allowing normalisation of pregnancy when HIV is diagnosed and treated early.

